# Transformations in the Ti-6Al-4V Alloy Studied Using Dilatometry Supported by Acoustic Emission

**DOI:** 10.3390/ma17246260

**Published:** 2024-12-21

**Authors:** Małgorzata Łazarska, Janusz Musiał, Tomasz Tański, Zbigniew Ranachowski

**Affiliations:** 1 Faculty of Material Engineering, Kazimierz Wielki University, Chodkiewicza 30, 85-064 Bydgoszcz, Poland; 2 Faculty of Mechatronics, Kazimierz Wielki University, Mikołaja Kopernika 1, 85-074 Bydgoszcz, Poland; janusz.musial@ukw.edu.pl; 3 Institute of Engineering Materials and Biomaterials, Silesian University of Technology, Konarskiego 18A, 44-100 Gliwice, Poland; tomasz.tanski@polsl.pl; 4 Institute of Fundamental Technological Research, Polish Academy of Sciences, Pawińskiego 5B, 02-103 Warszawa, Poland; zranach@ippt.pan.pl

**Keywords:** Ti-6Al-4V alloy, microstructure, dilatometric tests, acoustic emission (EA)

## Abstract

This paper presents the results of research on the kinetics of transformations in the two-phase (α + β) Ti-6Al-4V alloy. The transformation start and end temperatures during heating at different rates were determined using a dilatometer. A modified dilatometer was employed, equipped with an acoustic emission measurement apparatus and software enabling the assessment of sample dimensional changes during heating and cooling. The results were obtained in the form of dilatometric curves. Additionally, the occurrence of the transformation was confirmed by acoustic emission signals. In the study of the Ti-6Al-4V alloy, acoustic emission refers to the application of this non-destructive technique to monitor the alloy’s behavior during thermal processes. As the temperature increased, regardless of the heating rate, the α→β transformation was observed to occur after exceeding 900 °C. Within the transformation range, acoustic emission signals were recorded. Moreover, it was found that the applied research methods enabled the identification of signal components originating from the transformation. The application of acoustic methods in the analysis of phase transformations opens new possibilities for their use in industry.

## 1. Introduction

In recent years, titanium and its alloys have found extensive applications across various industries, particularly as components in machinery and equipment. Titanium and its alloys are materials with exceptional properties, making them highly valued in various fields. The lightweight nature of titanium, combined with its high strength, corrosion resistance, and biocompatibility, makes it ideal for use in industry, technology, and medicine. However, the widespread use of titanium alloys, especially in the production of machine parts, is somewhat limited by their unfavorable tribological properties, such as a tendency toward galling [1]. To improve these properties, titanium alloys undergo various surface treatments [2,3,4]. These processes aim to enhance wear and galling resistance, enabling the more efficient utilization of titanium in industrial applications. Thanks to these modifications, titanium can find wider applications in demanding conditions, where durability and reliability are key.

Recently, the advancement of measurement techniques has led to the development of various research methods. This has made it possible to conduct detailed chemical and physical analyses of materials with diverse properties [5]. Dilatometric studies, due to their versatility and specificity, have gained particular recognition and are applied in various fields of science and industry. In materials engineering, dilatometry is used for designing new materials, studying their degradation, measuring dimensional changes, recording phase transformations, determining the coefficient of thermal expansion, and evaluating the mechanical and thermal properties of materials [5]. Dilatometry is an effective method in polymer research, particularly in the context of analyzing phase transformations and degradation and evaluating thermomechanical properties. Its application can provide essential information for designing and optimizing the use of polymer materials in industry [6,7]. In the metallurgical industry, dilatometry is employed to analyze thermal processes, characterize phases, and optimize heat and thermo-chemical treatments. In the medical industry, it is used to analyze the behavior of biomaterials utilized in implantology under various temperature conditions. Dilatometry is an indispensable research tool in situations where it is crucial to observe the response of materials to temperature changes [8,9,10].

The two-phase Ti-6Al-4V alloy is the most widely used high-strength titanium alloy. One of the most critical aspects is the control and optimization of the microstructure of this (α + β) alloy, enabling the achievement of desired properties in finished industrial products [11]. Properly selected heat treatment parameters ensure the formation of a specific microstructure. The heating and cooling rates have a significant impact on the resulting microstructural characteristics. In the case of a lamellar microstructure, key parameters include the thickness of the α layers at the primary β grain boundaries, the thickness and size of the α plates, and the colony size [12,13,14]. As the cooling rate increases, all these parameters decrease. Many researchers have applied heat treatment processes to the Ti-6Al-4V alloy to adjust and enhance its strength and ductility [15,16,17]. Efforts are currently focused on improving properties and optimizing thermal and design processes, as Ti-6Al-4V remains one of the most widely used titanium alloys in the industry.

The acoustic emission (AE) method is used in studies of material properties and structure, enabling the understanding of processes occurring within them [18,19,20]. The acoustic emission technique is widely employed for monitoring various engineering objects. The sources of acoustic emission signals are processes in which elastic waves propagate through the monitored object. Thus, signal sources may include processes associated with deformation or cracking mechanisms [21]. They may also involve crack formation and propagation, corrosion processes, and the oxidation of gases related to structural leaks.

The acoustic emission method has also been adopted in materials engineering. AE signals are generated as a result of the localized release of stored internal energy within the tested material. This is a non-destructive testing method that enables the analysis of various physical phenomena. Acoustic emission is used in the monitoring and analysis of phase transformations occurring in metals during heat treatment processes [22,23,24]. The sources of signals generated during thermal processes include the movement of dislocations due to plastic deformation, the formation of twins, and displacement-type phase transformations [16,17].

The study of transformations in Ti-6Al-4V titanium alloys using dilatometry and the highly sensitive acoustic emission method can significantly enhance the understanding of the phenomena involved. The research results can contribute to improving heat treatment processes, which, in turn, will positively impact the properties of high-strength and durable materials.

## 2. Research Methodology

Phase transformations occurring during the heating of the two-phase Ti-6Al-4V titanium alloy were studied. The chemical composition of the alloy is presented in Table 1.

**Figure 1:**
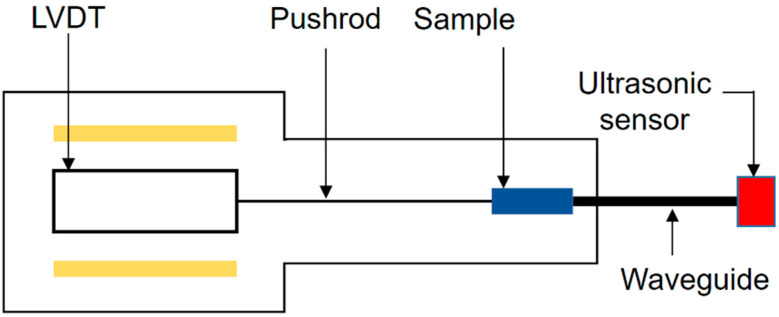
Schematic diagram of the measurement device for dilatometric tests. Linseis dilatometer with modifications, including a waveguide and an ultrasonic sensor for capturing AE signals. LVBT—Linear Variable Differential Transformer.

A quenching dilatometer (LINSEIS L75HX, Selb, Germany) integrated with an apparatus for acoustic emission signal measurement was used for the dilatometric tests. The device enabled the measurement of absolute sample length changes and the coefficient of thermal expansion as a function of temperature and time. Its design ensures the highest accuracy, repeatability, and stability of measurements. The dilatometer consisted of a measuring and control unit, as well as a computer system equipped with precise software for recording and analyzing results, presented as dilatometric curves. The dilatometer underwent mechanical modifications. In the measuring system, at the location designated for the test sample, a waveguide was installed to capture impulses during testing. An ultrasonic sensor of WD type (20–900 kHz) (Physical Acoustic Corporation, West Windsor Township, NJ, USA) was mounted at the other end of the waveguide to record acoustic emission signals. The acoustic sensor was then connected to an analyzer. A prototype analyzer for recording acoustic emission signals, developed at the IPPT PAN in Warsaw (Institute of Fundamental Technological Research, Polish Academy of Sciences), was used. The device is equipped with a low-noise amplifier and both high-pass and low-pass filters. The analyzer was designed to work with the broadband AE sensor from Physical Acoustics. The waveguide was made of austenitic steel, which does not generate acoustic emissions during heat treatment, as confirmed by conducted tests. A diagram of the modified dilatometer capable of measuring acoustic emission signals is shown in Figure 1.

Samples for dilatometric tests were prepared from the Ti-6Al-4V alloy in the form of cylinders with a diameter of 10 mm and a length of 18 mm. Each sample was subjected to controlled heating using the Linseis L75HX 1000 dilatometer. This advanced device features a modular design that enables precise measurements of material dilatation over a wide temperature range. The device consists of a resistance furnace, a measurement mechanism, a sample mounting system, and a cooling system. The samples were heated at different rates of 8 °C/min, 12 °C/min, and 14 °C/min. The material was heated to 1000 °C and then air-cooled at a rate of 50 °C/min. The dilatometer monitors changes in the length of a sample during controlled heating and cooling. The sample, in the form of a rod, is placed in a special holder of the dilatometer and subjected to controlled heating. A highly sensitive LVDT (Linear Variable Differential Transformer) sensor is used to detect changes in the sample’s length (expansion and contraction). This sensor records the movement of the plunger in contact with the sample with micrometric accuracy. Software is employed to visualize dilatometric curves and analyze the material’s properties.

The modified dilatometer operates similarly to a commercial dilatometer. The difference lies in the installation of a 4H13 steel waveguide (non-acoustic emission generating) in the sample holder inside the dilatometer. The waveguide is mounted to make contact with the test sample during the experiment. On the other side of the waveguide, a broadband WD sensor (20–900 kHz) is installed. The sensor is then connected to an analyzer that records AE signals.

**Figure 2:**
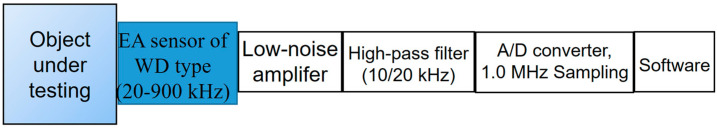
Schematic diagram of the AE signal recording device (analyzer: the prototype was developed at IPPT PAN, the AE sensor is a WD-type sensor from Physical Acoustics Corporation, and an ADLINK 9812 data acquisition card is used).

During the recording of results in the form of dilatometric curves, acoustic emission signals originating from phase transformations during the heat treatment of the titanium alloy were also captured. The signal recording was performed using a custom measurement setup. The schematic of the device for recording acoustic emission signals is shown in Figure 2.

During the design of the setup, it was taken into account that the effect of the dependence of the AE signal energy on the signal generation location could be neglected at short distances from the transducer (less than 200 mm). An acoustic sensor was attached to the end of the waveguide located outside the dilatometer, which was then connected to an analyzer. Elastic waves generated during the phase transformation reach the surface of the material. Then, via a waveguide in contact with the sample, they are received by the acoustic sensor. Subsequently, they are converted into an electrical signal using a piezoelectric transducer. For an AE analysis, it is necessary to measure the amplitude and duration of pulses ranging from microseconds to tenths of a second. The measurement setup used for recording and analyzing AE signals consisted of a broadband acoustic emission sensor of the WD type, a single-channel acoustic emission analyzer with a bandwidth of 1–1000 kHz, built-in active filters, and a root mean square (RMS) converter output. Signal registration, received by the piezoelectric transducer, was carried out using the analyzer. The analyzer enabled cyclic recording of approximately 100 ms from each subsequent second of the signal generated during phase transformations in the titanium alloy. The noise level in the operating frequency band did not exceed 50 microvolts RMS (peak-to-peak). The amplifier allowed signal amplification in the range of 20 to 60 dB. A high-pass filter connected to the amplifier output was used to eliminate background noise from the measurement system. The recorded signal, in wav format, was further processed and analyzed. Using Spectrum software (prototype version), the signal was converted into a spectrogram, a time–frequency plot. The spectrogram was generated using the Short Time Fourier Transform (STFT) algorithm with a Hamming window. The resolution of the plot was 0.6 kHz/1 s, with a maximum rendered frequency of 500 kHz.

## 3. Results of the Research

**Figure 3:**
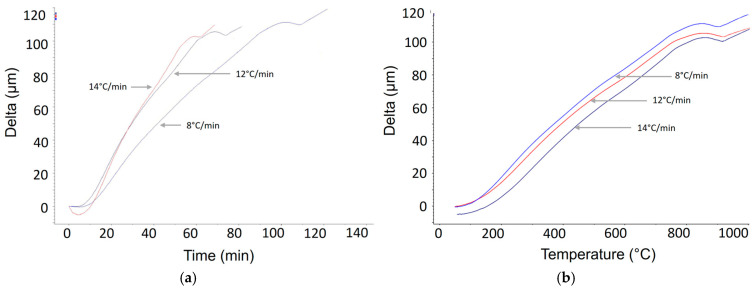
Dilatometric curve from the test during heating of the Ti-6Al-4V alloy: different heating rates of 8 °C/min, 12 °C/min, and 14 °C/min. (**a**) the sample length changes as a function of time, (**b**) the sample length changes as a function of temperature.

Dilatometric tests were performed, and as a result, dilatometric curves were obtained. The graphs presented in Figure 3 show the transformation of the Ti-6Al-4V alloy during its heating.

The thermal dilatometric curves shown in Figure 3 illustrate the transformations occurring within a specific temperature range during the heating of the Ti-6Al-4V alloy at different rates. It can be observed that the changes in thermal expansion occur similarly for all heating rates. The beginning of the change in the direction of the dilatogram at a heating rate of 8 °C/min was observed at a temperature of approximately 750 °C and after about 90 min from the start of the process. At a rate of 12 °C/min, the curve deviates from linearity at 760 °C (70 min); at 14 °C/min, it is 760 °C (52 min). The transformation ends at a temperature of approximately 910–940 °C.

At these temperatures, structural changes begin to occur in the material. In all three graphs, the transformations occur similarly, differing only in the rate of occurrence. The slower the heating rate of the titanium alloy, the slower the α + β → β transformation proceeds. During slow heating, the initial stage involves the formation of β-phase nuclei at the grain boundaries due to a shearing mechanism [25,26]. This is followed by the growth of β-phase nuclei driven by elemental diffusion mechanisms, and finally, their expansion in the final stage. During the heating process, the β-phase transformation occurs when the elemental concentration reaches a critical value. The β-phase transformation, which occurs in materials such as metal alloys, is related to the transition between different crystal structures under the influence of temperature and element concentration. This phenomenon is often associated with the so-called critical temperature or critical concentration value that triggers the phase change. In the case of some metal alloys, such as titanium alloys, the β-phase transformation can occur when the concentration of the alloying element reaches a value at which the material’s structure transitions from one form to another. In the context of thermal processes, such as heating the material, the β-phase transformation can occur due to an increase in temperature, with the critical concentration value of the alloying element determining the moment of this change. The critical concentration value may vary depending on the chemical composition of the material and technological process parameters, such as heating time and the presence of other impurities. The dilation coefficient was also calculated for all applied heating rates within the temperature range of 20–800 °C. The dilatation ratios for different heating rates are presented in Table 2.

**Figure 4:**
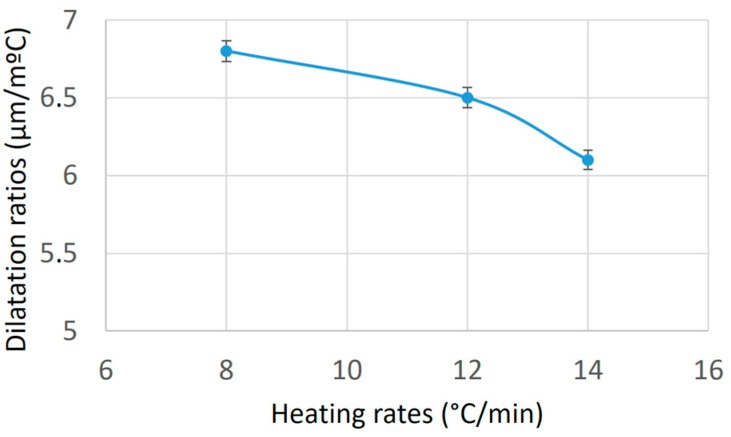
The correlation between the dilatation ratios and different heating rates.

Additionally, Figure 4 presents a graph correlating the dilatation ratios at different heating rates of the Ti-6Al-4V alloy. It can be observed that the dilatation ratio changes depending on the heating rate. As the heating rate increases, the dilatation ratio decreases. The increase in temperature is progressively greater in relation to the increase in the sample’s length.

**Figure 5:**
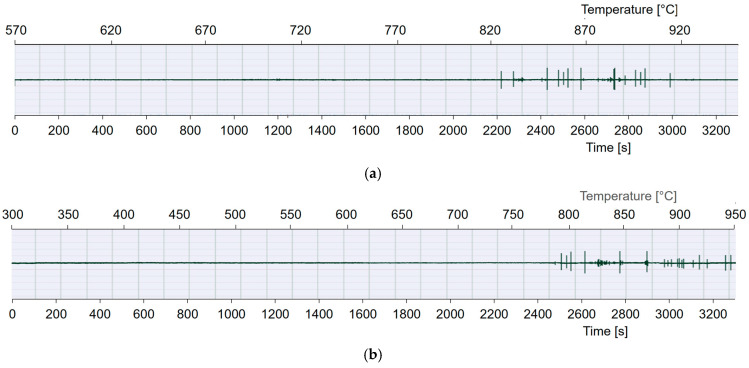

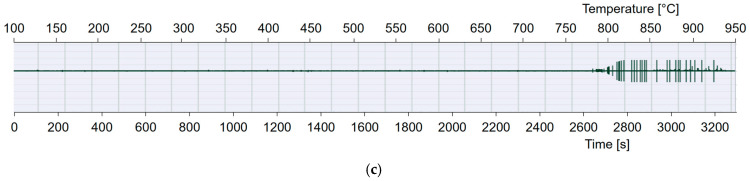
Comparison of the time courses of the RMS value of the AE signal recorded during the heating of the Ti-6Al-4V alloy at different heating rates of (**a**) 8 °C/min, (**b**) 12 °C/min, and (**c**) 14 °C/min.

AE signals generated during the heat treatment of the Ti-6Al-4V alloy, recorded using specialized equipment connected to an ultrasonic transducer, are shown in Figure 5a–c.

**Figure 6:**
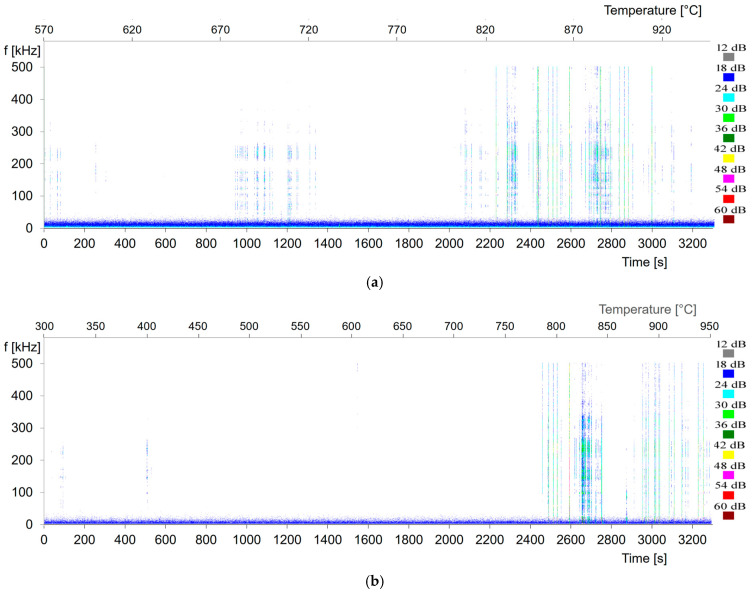

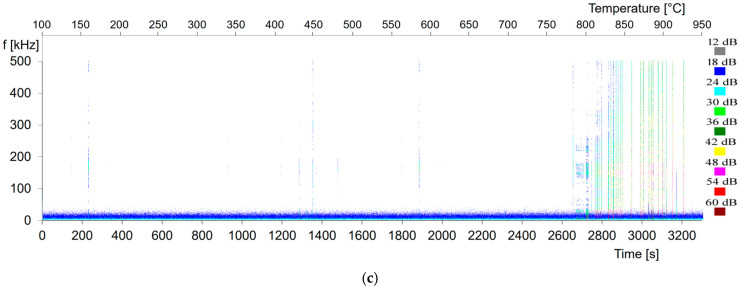
Spectrograms of the signal energy spectrum recorded during the heating process of the Ti-6Al-4V alloy at different heating rates of (**a**) 8 °C/min, (**b**) 12 °C/min, and (**c**) 14 °C/min; horizontal axis—signal recording time, vertical axis—signal frequency.

The results of AE measurements and the presented spectral images of the acoustic emission signal originating from the α + β → β transformation for the Ti-6Al-4V alloy are shown in Figure 6a–c.

The results of the acoustic emission signals and spectral images indicate that during the heat treatment of the investigated material, a physical phenomenon occurred that generated acoustic signals. In studies utilizing AE in solid materials, metals, and their alloys, several research directions are commonly observed. The most notable includes the investigation of atomic structure reconfiguration, which is closely linked to impurity diffusion. Another focus is the study of dislocations, specifically their density, multiplication, and lattice geometry. The presented images show that no peaks are observed at the beginning of the heating process, corresponding to the acoustic background level. The level of acoustic signals is relatively low, despite the intensive α + β → β transformation, growth, and expansion of β-phase nuclei. The transformation progresses through the increase in β-phase volume via the displacement of the α/β interphase boundary. The measuring device allows for the cyclic recording of approximately 100 milliseconds of data from every subsequent second of the signal generated during the phase transformations of metal alloys. These conditions enable prolonged signal recording, up to 3200 s. However, longer recordings result in some data loss, which is why the signal characteristics shown in the figures above are less intense. Nevertheless, they indicate the start and completion of the phase transformation. The dominant spectral range of the AE signals generated during the tests falls within 100–300 kHz. The maximum spectral density is observed in the frequency range of 180–200 kHz. The recording of the acoustic emission (AE) signal began 53 min before the end of the phase transformation. For the heating rate of 8 °C/min, recording started 65 min after the beginning of the sample heating, at approximately 570 °C, continuing until 950 °C. For the heating rate of 12 °C/min, recording started 26 min after the heating began, at approximately 300 °C. Similarly, for the heating rate of 14 °C/min, it began after 14 min, from 100 °C to 950 °C.

For the LINSEIS L75HX dilatometer, the error analysis of measurements encompasses the hardware characteristics, the properties of the samples being studied, and the experimental conditions. Typical errors mainly arise from selecting an inappropriate reference material for the temperature range of the measurements. Additionally, inaccuracies may occur in reading the calibration curves of the displacement sensor. Proper calibration of the device is crucial to minimizing measurement errors. Calibration is performed systematically using reference materials such as quartz or Al_2_O_3_. The accuracy of thermal expansion measurements for the LINSEIS L75HX is estimated to be ±0.1% under standard conditions. The resolution of the displacement sensor is within the nanometer range, and the repeatability is at the level of ±150 nm. Moreover, appropriate sample preparation can also reduce errors; in particular, mechanical stresses during sample mounting should be avoided.

**Figure 7:**
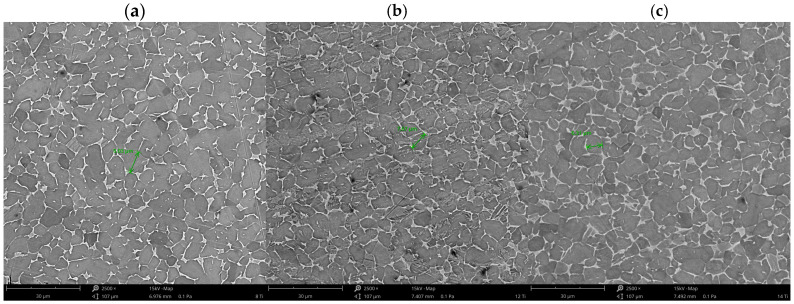
Microstructure images of Ti-6Al-4V samples after chemical etching in Kroll solution: (**a**) heating 8 °C/min, rapid cooling; (**b**) heating 12 °C/min, rapid cooling; and (**c**) heating 14 °C/min, rapid cooling.

The microstructural analysis of the Ti-6Al-4V alloy subjected to heat treatment was performed using a Phenom XL scanning electron microscope (Thermo Fisher Scientific, Waltham, MA, USA). Microscopic studies enable the comparison of microstructural images after heat treatment and etching, surface condition analysis, and morphology evaluation. The results of the microstructural studies are presented in Figure 7a–c.

The microstructure images reveal isometric grains with distinct boundaries, indicating dynamic recrystallization during heating. The microstructure contains acicular martensitic structures resulting from the cooling of the β phase. Both the α phase and remnants of the β phase are visible.

## 4. Discussion

The rapid development of technology in all fields provides increasing opportunities for studying metals. This progress enables the production of alloys with increasingly diverse and superior properties. The challenges in identifying phase transformations during the heat treatment of metals and their alloys arise from the complexity of the physical processes occurring within the volume of the material being studied. Acoustic methods can effectively complement dilatometric studies used to determine phase transformations. The structure of metals and alloys plays a key role in their properties. The conducted research demonstrated that the acoustic signature results from structural changes. The processes occurring during phase transformations generate signals of varying energy. It can be concluded that AE signals enable the identification of specific phenomena such as atomic structure reconfiguration, closely linked to impurity diffusion, and studies related to dislocations.

As a result of the dilatometric studies conducted, graphs of changes in the length of dilatometric samples as a function of temperature and time were obtained. Different heating rates (8 °C/min, 12 °C/min, and 14 °C/min) were applied to achieve various times for the beginning and end of the phase transformation α + β → β. Additionally, to monitor the beginning and end of the transformation, an additional, more sensitive research method, acoustic emission, was used. Dilatometric studies were conducted using a modified dilatometer equipped with a waveguide and an acoustic sensor. The dilatometer was custom-made by the manufacturer according to the design provided by the author of the study. The beginning of the change in the direction of the dilatogram at a heating rate of 8 °C/min was observed at a temperature of approximately 750 °C and after about 90 min from the start of the process. The transformation ended at a temperature of 940 °C. The beginning of the change in the direction of the dilatogram at a heating rate of 12 °C/min was observed at a temperature of approximately 760 °C and after about 70 min from the start of the process. The transformation ended at a temperature of 930 °C. In contrast, the beginning of the change in the direction of the dilatogram at a heating rate of 14 °C/min was observed at a temperature of approximately 760 °C and after about 52 min from the start of the process. The transformation ended at a temperature of 910 °C. Additionally, real-time monitoring of the transformation was conducted using acoustic emission. However, due to the limitations of the recording device, the analyzer was activated 53 min before each sample reached 950 °C. Various times of occurrence of the phenomenon were observed in the signal and on the spectrograms. It can be noted that the times of occurrence and duration of the acoustic emission signal are close to the onset time of the transformation on the dilatometric curve. However, considering that acoustic emission is a very sensitive and accurate research method, it may register the initial phenomena that initiate the transformation. This relates to the mechanism of the transformation itself.

The acoustic emission signals observed during the phase transformation range indicate phenomena that initiate the transformation. The moment when the curve loses its linear character does not correspond to the initiation of the acoustic signal, which becomes noticeable only when the transformation is at a more advanced stage. In dual-phase titanium alloys undergoing the α + β → β transformation, the β phase from the initial structure is present. The β phase may act as the first element or nucleus that plays a key role during the movement of elements from the α-phase (stabilizing the β phase) to the α/β interphase boundaries. The absence of acoustic emissions at the beginning of the transformation can be attributed to its slow diffusional mechanism. The diffusion process and atomic reorganization lead to the generation and annihilation of dislocations, which emit AE pulses. Changes in the crystal structure and differences in the thermal expansion of phases can lead to local stresses, emitting acoustic signals. Grain boundaries of the phases are the sites where the transformation initiates. Their migration and the growth of the β phase also generate acoustic emissions.

The course of transformation during heating depends on several factors, such as temperature, time, and the chemical composition of the alloy. The size and geometry of the sample, in turn, influence the rate of structural changes. Longer heat treatment durations allow for a more complete transformation, providing sufficient time for the phase transformation to occur in an equilibrium manner. The Ti-6Al-4V alloy has low thermal conductivity, which means that temperature gradients within the material can be significant during rapid heating.

The intensity of acoustic emission depends on the amount of material undergoing transformation and the rate of the process. The transformation intensity over time during the α + β→β transition is an important aspect. The α + β → β transformation generates low-to-moderate-intensity signals in a continuous manner, especially at temperatures near the β phase. Under rapid heating conditions, the transformation occurs more abruptly, resulting in more intense acoustic emission pulses.

The structure of the resulting alloy is characterized by the presence of acicular martensitic structures formed as a result of rapid cooling from the β phase. Both the α phase and residual β phase are visible. The martensitic transformation results from atomic displacements, lattice distortions of the solid solution, and the formation of twins. No grain fragmentation was observed, which is attributed to low stresses and deformations. The structure of titanium alloys can be shaped through cyclic heat treatment.

In the recorded AE signal, events of various energies were detected, indicating phase transformations occurring in the Ti-6Al-4V alloy during this time. The level of acoustic signals is relatively low, despite the intensive α + β → β transformation and the growth and expansion of β-phase nuclei. Cooling rates did not significantly influence the intensity or duration of the transformation. The transformation progresses through an increase in β-phase volume as a result of the displacement of the α/β interphase boundary. It can be observed that the change in thermal expansion occurs similarly at all tested heating rates. In the heat-treated Ti-6Al-4V alloy, AE signals are observed during phase transformations. However, not all alloys exhibit clear and effective sources of these signals. In cases where phase transformation involves very slow, diffusion-based atomic movement, the energy of the AE signal is very low. It is important to note that the mechanism of this phenomenon is not fully understood, and tracking changes in AE signals during phase transformation may provide significant research insights. Although AE studies have been conducted by various researchers focusing on metals, further research and analysis should be pursued [27,28,29].

Another approach known in the literature related to thermal processes involves improving the Ti-6Al-4V alloy using various welding techniques [30]. Additionally, the ESD (Electro-Spark Deposition) method, which involves applying thin coatings [31], leads to changes in materials. These processes lead to changes in the structure and mechanical properties of the treated materials.

The study of transformations occurring in Ti-6Al-4V alloys using dilatometry and acoustic emission is crucial for better understanding the mechanisms responsible for structural changes and the properties of these materials. The dilatometry method allows for precise monitoring of dimensional changes in the sample during thermal cycles, enabling the identification of characteristic temperatures for phase transformations. In turn, acoustic emission, as a highly sensitive research method, allows for detecting dynamic phenomena such as cracking, phase transformations, or stress relaxation in real time.

The results of these studies can significantly contribute to optimizing heat treatment processes, such as annealing or quenching, which directly affect the final properties of the materials. A better understanding of the phenomena enables the design of more efficient and predictable production processes, thereby increasing the performance and reliability of components made of Ti-6Al-4V, widely used in the aerospace, medical, and automotive industries.

## 5. Conclusions

The results of the AE signal analysis validated the relevance of the undertaken investigations. It can be assumed that a more detailed understanding of the spectral characteristics of recorded signals will enable the more precise identification of microstructures. The analysis of emissions induced by phase transformations could in the future be enriched with advanced computational tools, such as digital signal analysis algorithms. Utilizing methods to monitor the start and end of phase transformations may contribute to the optimization of industrial processes in the future.

From the conducted research on heating the Ti-6Al-4V alloy at various rates using research methods such as dilatometry and acoustic emission, the following conclusions can be drawn:

1. During the heating of the Ti-6Al-4V alloy, dilatometric studies reveal the occurrence of the α + β → β transformation on the dilatometric curves.
2. The results, in the form of AE signals and spectrograms, similarly indicate the occurrence of phase transformations, consistent with the dilatometric studies.
3. The start and end times of transformations in the examined alloy depend on the heating temperature of the Ti-6Al-4V alloy.
4. The acoustic emission method can be effectively used to assess the kinetics of complex phase transformations occurring during the heat treatment of metals and their alloys.
5. Monitoring the phase transformation process may facilitate the production of materials with better performance properties in the future.
6. Methods enabling the monitoring of the start and end of phase transformations using acoustic emission could find applications in industry.

## Figures and Tables

**Table 1:** Chemical composition of Ti-6Al-4V alloy, elemental content in wt.%.

Ti	Al	V	Fe	O	C
Remainder	6.0	4.1	0.2	0.18	0.04

**Table 2:** Dilatation ratios for different heating rates.

Heating Rates (°C/min)	8 °C/min	12 °C/min	14 °C/min
Dilatation ratios (μm/mºC)	6.8	6.5	6.1

## Data Availability

The original contributions presented in this study are included in the article. Further inquiries can be directed to the corresponding author.
